# Simulating the Influence of Conjugative-Plasmid Kinetic Values on the Multilevel Dynamics of Antimicrobial Resistance in a Membrane Computing Model

**DOI:** 10.1128/AAC.00593-20

**Published:** 2020-07-22

**Authors:** Marcelino Campos, Álvaro San Millán, José M. Sempere, Val F. Lanza, Teresa M. Coque, Carlos Llorens, Fernando Baquero

**Affiliations:** a Department of Microbiology, Ramón y Cajal University Hospital, IRYCIS, Madrid, Spain; b Biotechvana, Valencia Technological Park, Paterna, Spain; c Valencian Research Institute for Artificial Intelligence (VRAIN), Universitat Politècnica de València, Valencia, Spain; d National Center for Biotechnology (CNB-CSIC), Madrid, Spain; e Network Research Center for Epidemiology and Public Health (CIBERESP), Madrid, Spain; f Bioinformatics Support Unit, IRYCIS, Madrid, Spain

**Keywords:** antibiotic resistance, complex systems, computational biology, computer modeling, conjugation, ecosystems, membrane computing, plasmids

## Abstract

Bacterial plasmids harboring antibiotic resistance genes are critical in the spread of antibiotic resistance. It is known that plasmids differ in their kinetic values, i.e., conjugation rate, segregation rate by copy number incompatibility with related plasmids, and rate of stochastic loss during replication. They also differ in cost to the cell in terms of reducing fitness and in the frequency of compensatory mutations compensating plasmid cost. However, we do not know how variation in these values influences the success of a plasmid and its resistance genes in complex ecosystems, such as the microbiota. Genes are in plasmids, plasmids are in cells, and cells are in bacterial populations and microbiotas, which are inside hosts, and hosts are in human communities at the hospital or the community under various levels of cross-colonization and antibiotic exposure. Differences in plasmid kinetics might have consequences on the global spread of antibiotic resistance. New membrane computing methods help to predict these consequences. In our simulation, conjugation frequency of at least 10^−3^ influences the dominance of a strain with a resistance plasmid. Coexistence of different antibiotic resistances occurs if host strains can maintain two copies of similar plasmids. Plasmid loss rates of 10^−4^ or 10^−5^ or plasmid fitness costs of ≥0.06 favor plasmids located in the most abundant species. The beneficial effect of compensatory mutations for plasmid fitness cost is proportional to this cost at high mutation frequencies (10^−3^ to 10^−5^). The results of this computational model clearly show how changes in plasmid kinetics can modify the entire population ecology of antibiotic resistance in the hospital setting.

## Abstract (precis)

Bacterial plasmids harboring antibiotic resistance genes are critical in the spread of antibiotic resistance. It is known that plasmids differ in their kinetic values, i.e., conjugation rate, segregation rate by copy number incompatibility with related plasmids, and rate of stochastic loss during replication. They also differ in cost to the cell in terms of reducing fitness and in the frequency of compensatory mutations compensating plasmid cost. However, we do not know how variation in these values influences the success of a plasmid and its resistance genes in complex ecosystems, such as the microbiota.

## INTRODUCTION

Plasmid kinetics are widely assumed to necessarily influence the spread of antibiotic resistance genes in bacterial populations and ecosystems (1–10). The main parameters that affect plasmid kinetics are as follows: (i) the rate of plasmid conjugation/transfer (the rate at which a bacterial cell harboring a conjugative plasmid [donor] transfers this plasmid to a recipient cell), (ii) the segregation rate due to plasmid incompatibility (considering the number of plasmid genome copies that are stably maintained in a bacterial cell), (iii) the plasmid cost (the reduction in growth rate of the host cell imposed by the presence [and transfer] of a plasmid), (iv) the rate of plasmid cost compensation (measuring the effect of mutations reducing plasmid cost), (v) the frequency of mutational events in the plasmid or bacterial genome, and (vi) the rate of plasmid loss (the rate at which plasmids are lost during the bacterial replication process).

However, the effects of these changes on the kinetics of plasmid resistance genes among bacterial populations are necessarily influenced by numerous other factors acting in actual biological ecosystems, such as the intestinal microbiota. Of these factors, our previously published studies on modeling by membrane computing (11–13) considered the following: the ecosystem’s bacterial composition, the density and replication rate of cells in each species, their mutation frequencies, the presence of chromosomal resistance genes, the selection intensity of resistant organisms due to differing antibiotic exposures, the elimination of susceptible bacterial populations by antibiotic treatment, the transmission of resistant bacteria among human hosts in hospital settings under differing admission-discharge rates, cross-colonization, and exposure to various antibiotics, as well as the influence of antibiotic resistance in nonhospitalized individuals who eventually pass through the hospital environment.

Experimentally investigating the influence of changes in plasmid kinetic parameters is extremely difficult and perhaps impossible under real-world conditions. The research involves a complex, multilevel, multiparametric, and interactive landscape, involving genes, cells, populations, communities, hosts, and factors that influence transmission and selection. However, this problem can be approached using novel computational models integrating within-host and between-host modeling (14, 15). Multilevel membrane computing models can provide an ecosystem-like framework composed of discrete independent but interactive units mimicking biological ones in a multihierarchical landscape of nested entities (e.g., genes inside plasmids, plasmids inside bacteria, bacteria inside microbiota, microbiota inside hosts, and hosts inside the hospital and interacting with the community) (12).

Membrane computing is conceptually based on complex biological systems, which are characterized by structured nested biological entities that can be conceptualized as separated by “membranes” (16, 17). Each gene, plasmid, cell, species, population, host, and also the compartment where the host is located (such as the community or hospital) is surrounded by “computational membranes” forming a multilevel nested structure that can be studied by devices such as membrane systems or P systems (12, 13). The P system applied in this study mimics the complex biological landscapes in the computer world. For instance, a plasmid is a computational object (a discrete entity) and has a “membrane” as a symbol of being something independent. But the bacterial cell is also a discrete entity and, as such, is individualized by a computational membrane (in that case it also has a real membrane), and the patient is an independent individual inside a computational membrane. In membrane computing, each of the nested “membrane-surrounded entities” can independently replicate, propagate, become extinct, transfer into other membranes, exchange informative material according to flexible rules, mutate, and be selected by external agents (13). In this work, this type of computational model helps simulate the combined effects of changes in the various parameters influencing the spread of antibiotic resistance plasmids, the key drivers of the dissemination of antibiotic resistance (18–20). The aim of this research is to explore how changes in these parameters influence the spread of antibiotic resistance genes located in plasmids, in bacterial populations, and in microbial communities composed of different bacterial species and which are the consequences of this spread. We also explore how these changes and their effects determine the frequency of various clones or species that harbor plasmids with antibiotic resistance genes. A simplified representation of the elements introduced in the membrane computing system is presented in Fig. S1 in the supplemental material. The kinetic properties of plasmids involved in the spread of high-risk antibiotic-resistant bacterial clones have been studied in laboratory experiments (see “Basal plasmid kinetic values” in Material and Methods), but their consequences in the long-term evolution of antibiotic resistance in complex, variable, and hospitalized environments are only accessible by modeling.

In summary, in this manuscript, what is being modeled is a hospital with patients being admitted or released, these human hosts harboring bacterial communities, and then plasmid dynamics within bacteria. We present a number of case studies analyzing the influence of changing plasmid kinetic values on complex hierarchical dynamics of antibiotic resistance in a simulated hospital setting. Most of the computing experiments referred to in this study mimic an evolution of 4.5 years (40,000 one-hour steps), with this period of time roughly corresponding to “significant changes” in antibiotic resistance in most reports (21–24). To our knowledge, this is the first study that has addressed the effect of plasmid kinetic parameters on the structure of multilevel biological systems.

## RESULTS

The results presented in this section reflect how changes in factors determining plasmid kinetics (acquisition, dissemination, loss) influence the long-term spread of antibiotic resistance among Escherichia coli and Klebsiella pneumoniae in a hospital with fixed (but modifiable at the will of the model user) rates of patient admission-discharge, antibiotic exposure to different antibiotics with particular dosages acting on the host’s microbiota, and between-host transmission of microorganisms (25, 26). Also, the model illustrates how the abundance of these and other bacterial species, such as Enterococcus faecium, have different susceptibilities to ampicillin or are a minority in the gut, such as Pseudomonas aeruginosa. The results of the model clearly show how plasmid kinetics can modify the entire population ecology of antibiotic resistance in the hospital setting. The details and abbreviations employed in the model are provided in Materials and Methods. However, to facilitate understanding of the Results section, Table 1 is presented below.

**TABLE 1:** Description of abbreviations

Abbreviation	Definition	Note(s)
AbA	Antibiotic A	For example, aminopenicillins
AbC	Antibiotic C	For example, 3rd generation cephalosporins
AbF	Antibiotic F	For example, fluoroquinolones
AbAR	Plasmid-mediated resistance to AbA	For example, TEM-1
AbCR	Plasmid-mediated resistance to AbC	For example, ESBLs
AbF*R	Chromosomal fluoroquinolone resistance target mutation	For example, *gyrA* mutation
AbA*R	Intrinsic chromosomal gene conferring AbAR in K. pneumoniae	For example, SHV-1
PL1	Plasmid 1, originally in an E. coli population, with AbAR	For example, IncF plasmids but able to coexist in the same cell
PL3	Plasmid 3, originally in K. pneumoniae, AbCR and AbAR	For example, IncF plasmids but able to coexist in the same cell
Ec0	E. coli without plasmids, fully susceptible	Pink lines in figures
EcA	E. coli with PL1-AbAR	Red lines in figures
EcC	E. coli with PL3-AbCR-AbAR	Light fluorescent green lines in figures
EcF	E. coli with chromosomal AbF*R	Violet lines in figures
EcAC	E. coli with PL1 and PL3 AbAR-AbCR	Light blue lines in figures
EcAF	E. coli with PL1 AbAR and chromosomal AbF*R	Brown lines in figures
EcCF	E. coli with PL3 AbCR-AbAR and chromosomal AbF*R	Olive green lines in figures
EcACF	E. coli with PL1 AbAR, PL3 AbCR-AbCR, and AbF*R	Dark blue lines in figures

### Influence of plasmid conjugation rates.

**FIG 1:**
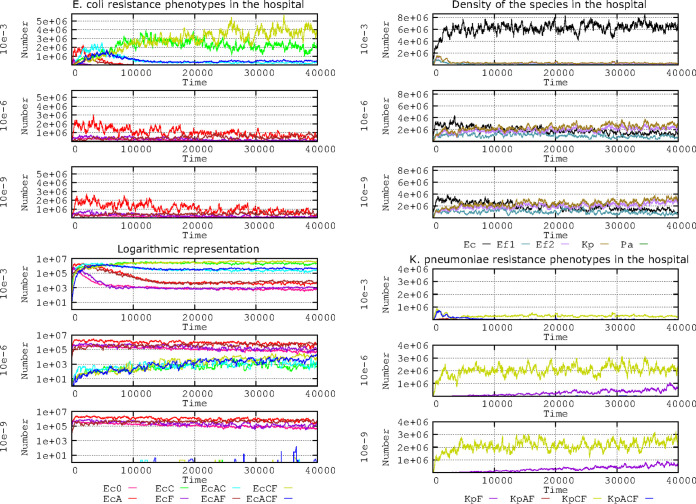
Influence of plasmid conjugation frequency (10^−3^, 10^−6^, 10^−9^) on the evolution of E. coli resistance phenotypes in the hospital. (Top left) Ec0, susceptible, no resistance plasmids (pink line); EcA, PL1-AbAR (red); EcC, PL3-AbAR-AbCR (light fluorescent green); EcF, AbF*R (violet); EcAC, PL1-AbAR plus PL3-AbAR-AbCR (light blue); EcAF, PL1-AbAR plus AbF*R (brown); EcCF, PL3, AbAR-AbCR plus AbF*R (olive green); EcACF, PL1-AbAR plus PL3-AbAR-AbCR plus AbF*R (dark blue). (Lower left) A logarithmic representation of the same resistance phenotypes. (Top right) Density of the species E. coli (black line), K. pneumoniae (olive green), ampicillin-resistant E. faecium (violet), and ampicillin-susceptible E. faecium (blue green). (Lower right) Detail of the evolution of K. pneumoniae (olive green) and ampicillin-resistant E. faecium (violet). Numbers in ordinates are expressed in hecto-cells (one unit = 100 cells in the microbiota). Note that the spread of populations with plasmid-mediated resistance correlates with plasmid conjugation frequency, and the effect is not visible with very low conjugation frequencies.

How to estimate plasmid transfer rates is not a trivial question (27). In this simulation, conjugation rates are expressed as the proportion of donor-recipient contacts that result in a random and reciprocal cell-to-cell E. coli-K. pneumoniae plasmid transfer in a given period per hour. For instance, 10^−6^ per hour indicates that 1 in 1 million donor-recipient contacts resulted in random and reciprocal cell-to-cell E. coli-K. pneumoniae plasmid transfers per hour. Conjugation rates widely differ in different plasmid-host combinations and also are heavily influenced by the abundance and coalescence of interacting bacterial populations (27–30). Our model included three conjugation rates (10^−3^, 10^−6^, and 10^−9^) applicable to the model plasmids PL1 and PL3 (not real plasmids; not to be confused with the plasmid vectors pL1 and pL3!) harbored by either E. coli or K. pneumoniae. These conjugation rates encompass the high, medium, and low horizontal transfer rates of many plasmids involved in the spread of antibiotic resistance in hospitals (31–36). The rate of spontaneous plasmid loss (segregation) was 10^−5^, and the mutation frequency for plasmid cost compensatory mutations was 10^−5^ (mutants reduced to one half of the cost of harboring plasmids). Other default values are as described in Materials and Methods. The results for the various E. coli phenotypes emerging from the plasmid transfer and mutational resistance to fluoroquinolones are presented in Fig. 1. A short-term effect on the E. coli population structure is only observed at high plasmid transfer rates (10^−3^) (Fig. 1, left column, first and fourth panels). A first selective burst of AbAR (red line) is followed by the AbAR-AbF*R phenotype (brown) (due to the mutational evolution to fluoroquinolone resistance of AbAR cells) and by the acquisition of PL1-AbAR by the AbF*R cells. The acquisition of PL3 from K. pneumoniae occurs almost simultaneously (now, AbAR-AbCR, light blue) and then from AbF*R E. coli (now, AbAR-AbCR-AbAR, AbF*R, dark blue). Subsequently, the predominant populations are AbF*R E. coli harboring PL3 (AbCR-AbAR) and AbF*R (olive green).

K. pneumoniae is critical at the start of the process by providing the plasmid PL3 (AbCR-AbAR) to E. coli; however, at high transfer rates, most plasmid transfers occur among the E. coli cells, which thereby acquire most of the antibiotic resistance benefits (Fig. 1, right column, top panel). With medium to low transfer rates, E. coli populations appear to remain stable and do not spread significantly in the human host population (Fig. 1, right column, panels 2 and 3). Interestingly, a logarithmic representation (Fig. 1, left column, panels 4 to 6) reveals remarkable differences at the 10^−6^ and 10^−9^ transfer rates with regard to the population structure. At both the 10^−6^ and 10^−9^ transfer rates, there is a steady preservation of the fully susceptible E. coli population (pink), AbF*R E. coli (violet) and that containing PL1-AbAR (red), and populations harboring PL1-AbAR and AbF*R (brown). At the 10^−6^ transfer rate, however, a small part of the E. coli population acquires the PL3 plasmid from K. pneumoniae (and later from E. coli/PL3), giving rise to a constant increase in the phenotypes AbCR-AbAR (green), AbCR-AbAR-AbF*R (olive green), and AbAR-AbCR-AbAR, AbF*R (dark blue).

Even at the 10^−9^ transfer rate, a few E. coli cells capture the PL3 plasmid but are unable to spread AbCR efficiently in the E. coli population. The long-term maintenance of K. pneumoniae with PL3 (olive green) is only assured at low transfer rates (10^−6^, 10^−9^), impairing the dominance of resistant E. coli populations (Fig. 1, right column, panels 5 and 6) given that, at high transfer rates, the plasmid PL1 from E. coli invades the K. pneumoniae cells, which might lose their resident PL3 plasmids due to an excess in plasmid copy number. In summary, the short-term selection of the E. coli phenotypes depending on plasmid acquisition only occurs for the 10^−3^ conjugation frequency of E. coli phenotype, resulting in the consequent increase in prevalence of E. coli species and reduction in K. pneumoniae cells. However, there are differences between the 10^−6^ and 10^−9^ conjugation frequencies; in the first case, there is a slow spread over time of plasmid-mediated resistance phenotypes, whereas at 10^−9^, the original population structure remains essentially constant.

### Influence of plasmid compatibility.

Plasmids can be divided into the following two groups according to their size and biology: small plasmids that are maintained at multiple copies per bacterial cell and large plasmids with low copy number (37). It is true that small multicopy plasmids can be mobilized by conjugation by coresident conjugative elements (mobilizable plasmids), but strictly speaking, conjugative plasmids (self-transmissible) belong to the “large” group and exist at a low copy number per cell. When two large conjugative plasmids share identical partitioning (*par*) loci, that may result in mutual interference and possible loss in a few generations (33, 36, 38). Plasmids typically have a limited number of copies. Relevant conjugative plasmids in the spread of antibiotic resistance have a low number of copies (a maximum of 1 to 3 per cell, maximum 10) (35). Our model is limited to plasmids PL1 and PL3, which may correspond to highly related conjugative plasmids with a low copy number, one to three replicon copies, such as IncF plasmids (33, 35). These plasmids are the most frequently involved in the spread of β-lactamases (PL1) and extended-spectrum β-lactamases (ESBL) (PL3) in hospital environments. IncF plasmids have the particularity of carrying multiple replicons (34), which produces an apparently paradoxical situation where plasmids from the same “incompatibility group” are frequently compatible. Inspired by the pervasive coexistence of antibiotic resistance conjugative IncF plasmids in clinical enterobacteria, we defined the plasmids PL1 and PL3 as belonging to the same incompatibility group but allowed the plasmids to coexist in the host bacterium. However, even belonging to the same incompatibility group, the plasmids have differences that may affect, to a certain extent, their copy number control, limiting the number of PL1 or PL3 replicons. That’s why we explored how changes in the total copy number influence different copy number combinations of PL1 and PL3.

**FIG 2:**
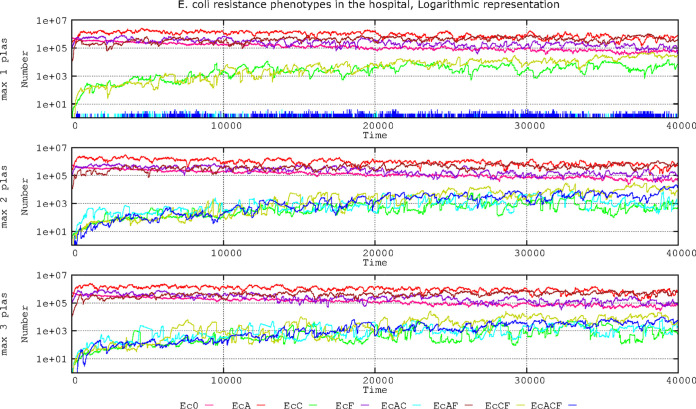
Effect of plasmid coexistence on the evolution of E. coli antibiotic resistance phenotypes in the hospital setting. Ec0, susceptible, no resistance plasmids (pink line); EcA, PL1-AbAR (red); EcC, PL3-AbAR-AbCR (light fluorescent green); EcF, AbF*R (violet); EcAC, PL1-AbAR plus PL3-AbAR-AbCR (light blue); EcAF, PL1-AbAR plus AbF*R (brown); EcCF, PL3, AbAR-AbCR plus AbF*R (olive green); EcACF, PL1-AbAR plus PL3-AbAR-AbCR plus AbF*R (dark blue). The rate of plasmid cost compensation was fixed at 10^−5^. Numbers in ordinates are expressed in hecto-cells (one unit = 100 cells in the microbiota). Note that if the cell is able to stably maintain one copy of PL1 and one or two copies of PL3 or one copy of PL3 and one or two of PL1, resistance to different antibiotics increases in time.

Therefore, we examined the effect of cells tolerating only 1 plasmid copy (PL1 or PL3), where any new incoming plasmid copy (either PL1 or PL3) is rejected or substituted by one of the resident copies. We also examined the condition where only 2 plasmid copies can coexist (2 PL1, 2 PL3, or 1 PL1 and 1 PL3) and when 3 plasmid copies can coexist (all 3 PL1, all 3 PL3, 2 PL1 plus 1 PL3, or 2 PL3 plus 1 PL1). The question is particularly relevant given that the presence of plasmid PL1 (AbAR) (originally located in E. coli) might prevent or influence the acquisition of plasmid PL3 (originally located in K. pneumoniae) or vice versa. Figure 2 shows the results of the effects of these maximum plasmid copy numbers in the population structure of E. coli in the hospital setting.

When a single copy of the plasmid replicon is tolerated in the cell (originally the plasmid-bearing E. coli cells have a copy of PL1), there is a progressive invasion of E. coli cells by the plasmid PL3 from K. pneumoniae, giving rise to an increase in the cephalosporin-resistant (and ampicillin-resistant) phenotype EcC (green line). Through mutation of these cells, the phenotype is expanded by acquisition of fluoroquinolone resistance (olive green). EcC can arise with even higher frequency following the acquisition of PL1 by AbF*R cells and then by displacement of PL1 by PL3 (initially in K. pneumoniae). The cells lose PL1 (AbAR); even if the PL1 plasmid is transferred, it does not remain in the cell (blue spikes at the bottom curve). With a maximum of 2 or 3 plasmid copies per cell, we can obtain a similar increase in resistant populations harboring both PL1 and PL3 with or without fluoroquinolone resistance (dark and light blue, respectively). In summary, as expected, if only one plasmid copy is maintained in the cell, the populations independently harboring PL1 and PL3 steadily increase over time. If two plasmids can coexist in the same cell, the density of the population carrying both plasmids also increases, but no major differences occur if the cell can stably maintain three plasmids. In any case, the emergence and progressive enrichment of populations able to harbor plasmids with different resistances are certainly important for the treatment of infected patients.

### Influence of random plasmid loss.

**FIG 3:**
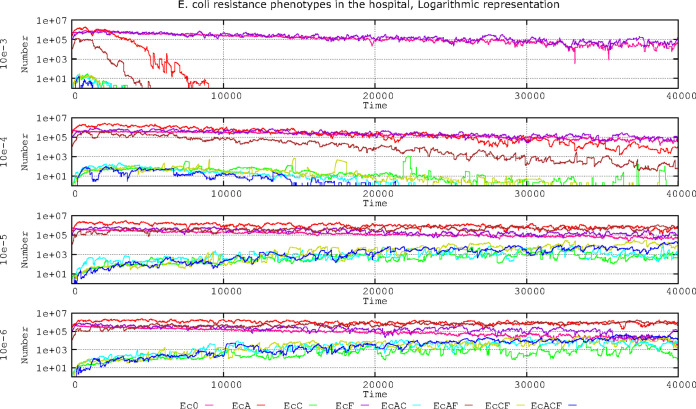
Influence of plasmid loss rates on the evolution of E. coli resistance phenotypes in the hospital environment. Ec0, susceptible, no resistance plasmids (pink line); EcA, PL1-AbAR (red); EcC, PL3-AbAR-AbCR (light fluorescent green); EcF, AbF*R (violet); EcAC, PL1-AbAR plus PL3-AbAR-AbCR (light blue); EcAF, PL1-AbAR plus AbF*R (brown); EcCF, PL3, AbAR-AbCR plus AbF*R (olive green); EcACF, PL1-AbAR plus PL3-AbAR-AbCR plus AbF*R (dark blue). Compensation rate for plasmid fitness costs was fixed at 10^−5^. Numbers are expressed in hecto-cells (one unit = 100 cells in the microbiota). Note that frequencies of plasmid loss equal to or higher than 10^−3^ impair the fixation of all E. coli lineages harboring resistance plasmids, but at lower rates of plasmid loss, beyond 10^−5^, E. coli populations with extended resistance phenotypes steadily increase over time.

In the process of cell division, the daughter cell receives at least one plasmid as a result of random diffusion influenced by multimer resolution systems (39). Conjugative plasmids have active partitioning systems to prevent nonbinomial segregation of plasmids into daughter cells. High-copy-number plasmids have random partitioning, and loss is mitigated by the high copy number. The rate of spontaneous plasmid loss in conjugative plasmids remains controversial (40). Our results (Fig. 3) indicate that at loss (random segregation) rates of 10^−3^, E. coli populations with plasmid PL1 or PL3 are not maintained beyond 8,000 steps (approximately 2 weeks). At segregation rates of 10^−4^, the most abundant plasmid (PL1 in E. coli, with AbAR, in red) is maintained. After an initial increase, however, the plasmid population containing PL3 steadily decreases because of the copy number incompatibility of PL3 with the dominant PL1 and due to the progressive reduction of K. pneumoniae with PL3, reducing the flow toward E. coli. Of course, cells with PL3 are more effectively selected by antibiotic exposure than those with PL1, but PL1 is comparatively more abundant than PL3 in the ecosystem; therefore, PL1 is maintained over the 40,000 steps.

E. coli cells containing plasmid PL3 can slowly increase in number only at 10^−5^ segregation rates. At 10^−6^, these cells reach the density of antibiotic-susceptible or PL1-containing cells. These results suggest that, at high plasmid segregation rates, the populations harboring the most abundant plasmids in the ecosystem have an advantage over populations with minority plasmids; however, if the loss of plasmids is relatively rare (such as 10^−6^), different plasmids might coexist in the population. Results with a 10^−7^ plasmid loss did not significantly differ from those of 10^−6^ (data not shown).

In summary, frequencies of plasmid loss equal or lower than 10^−3^ impair the fixation of all E. coli lineages harboring resistance plasmids. However, a frequency of 10^−4^ allows the long-term persistence of initially abundant plasmids such as PL1 and at much lower density populations with PL3. At plasmid loss rates of 10^−5^, and even more at 10^−6^, the E. coli populations with extended resistance phenotypes steadily increase over time.

### Influence of plasmid fitness costs.

**FIG 4:**
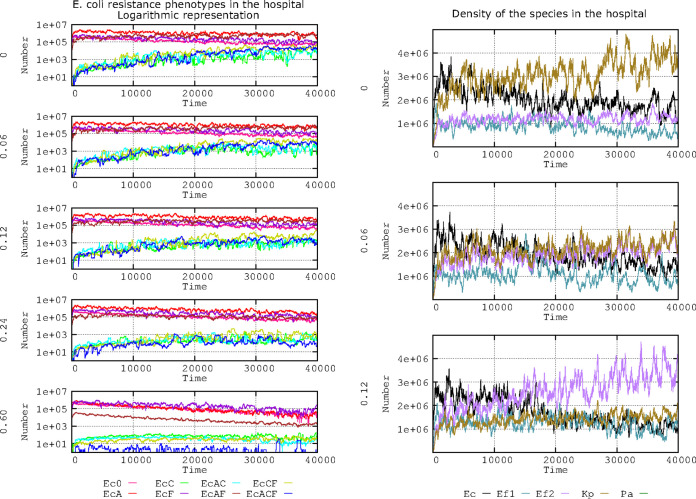
Influence of plasmid fitness cost on the evolution of E. coli antibiotic resistance phenotypes. The graphs in the left column show the effects for each fitness cost (0, 0.06, 0.12, 0.24, and 0.60). The lines indicate the following. Ec0, susceptible, no resistance plasmids (pink line). On the left, effects of no fitness cost (0), and 0.06, 0.12, 0.24, and 0.6 fitness costs. Ec0, susceptible, no resistance plasmids (pink line); EcA, PL1-AbAR (red); EcC, PL3-AbAR-AbCR (light fluorescent green); EcF, AbF*R (violet); EcAC, PL1-AbAR plus PL3-AbAR-AbCR (light blue); EcAF, PL1-AbAR plus AbF*R (brown); EcCF, PL3, AbAR-AbCR plus AbF*R (olive green); EcACF, PL1-AbAR plus PL3-AbAR-AbCR plus AbF*R (dark blue). The graphs in the right column show the influence of 3 fitness costs (0.0, 0.06, 0.12) on the species distribution in the simulated ecosystem as follows: E. coli (black line), K. pneumoniae (dark olive green), ampicillin-resistant E. faecium (violet), ampicillin-susceptible E. faecium (blue-green). Numbers in ordinates are expressed in hecto-cells (one unit = 100 cells in the microbiota). In the left column, note that the increase in plasmid fitness cost reduces the spread of multiresistant phenotypes, but costs below 0.12 have a small effect. In the right column, plasmid costs equal or superior to 0.06 produce a reduction in the frequency of K. pneumoniae.

Plasmid fitness costs imposed on the bacterial host are considered a factor that contributes to the spread of a particular plasmid and its genes (41–45). Several values for PL1 and PL3 plasmid fitness costs were included in the model to ascertain the effect of hosting these plasmids (or not) on antibiotic resistance and species composition. For reference, a plasmid fitness cost of 0.06 indicates a 6% reduced growth rate for the E. coli and K. pneumoniae strains harboring the PL1 or PL3 plasmid. We investigated the influence of a range of fitness costs in comparison with “no fitness cost” (fitness cost = 0). Default values included in this model were as follows: only 2 plasmids can coexist in a single cell, the rate of spontaneous plasmid loss is 10^−5^, and the mutation frequency to reduce 50% of the plasmid fitness cost is 10^−8^. The results are presented in Fig. 4.

Regarding E. coli phenotypes (Fig. 4, left column), the effect of increasing the plasmid fitness cost was to steadily reduce the number of strains harboring only PL3 (encoding AbCR) or PL3 and PL1, alone or in combination with PL1 (AbAR) or AbF*R (green, blue, olive green, dark blue lines, respectively). Only with a high plasmid fitness cost (0.60) was there a clear reduction of the predominant populations containing PL1 in the absence (red line) or presence (brown line) of chromosomal AbF*R, probably due to the maintenance of an effective plasmid transfer. Note that with high plasmid fitness cost, the population with only chromosomal mutation (not influenced by plasmid fitness cost [AbF*R]) rises to dominance (violet line) but tends to decrease slightly, possibly because the reduction in cell multiplication of plasmid-containing cells results in reduced cell densities and, therefore, decreases the possibility of emergence of cells with new AbF*R mutations.

Differences in plasmid cost, even comparing no cost with the 0.06 or 0.12 plasmid cost, might influence the species structure in the hospital ecosystem. At cost 0, K. pneumoniae with PL3 (dark olive green) steadily increases in frequency. K. pneumoniae with PL3 originally benefits from the AbAR (including AbA*R) and AbCR phenotypes and progressively acquires fluoroquinolone resistance, surpassing E. coli with PL1, with only the AbAR phenotype (black). At cost 0.06, the long-term dominance of K. pneumoniae is strongly reduced, but E. coli also decreases in frequency. With a cost of 0.12, E. faecium (in which PL1 and/or PL3 are naturally excluded due to their host range but with an AbA*R-AbC*R phenotype—due to AbAR and AbCR chromosomal penicillin-binding proteins [PBPs]; violet) tends to dominate. Thus, the cost of harboring a plasmid (decreasing cell growth rates) might influence the abundance and diversity of species present in the hospital.

In summary, the density of E. coli populations harboring plasmids over time certainly increases in inverse proportion to the plasmid fitness costs, with this effect less patent on abundant plasmids, where the loss is compensated by plasmid recovery by conjugational transfer. Costs equal to or higher than 0.12 produce a clear disadvantage for the long-term spread of populations with new resistant phenotypes dependent on plasmid acquisition; note that with high costs, abundant plasmids reduce, very slowly, their frequency in E. coli populations.

### Effect of changes in compensatory mutation frequency for plasmid fitness costs.

**FIG 5:**
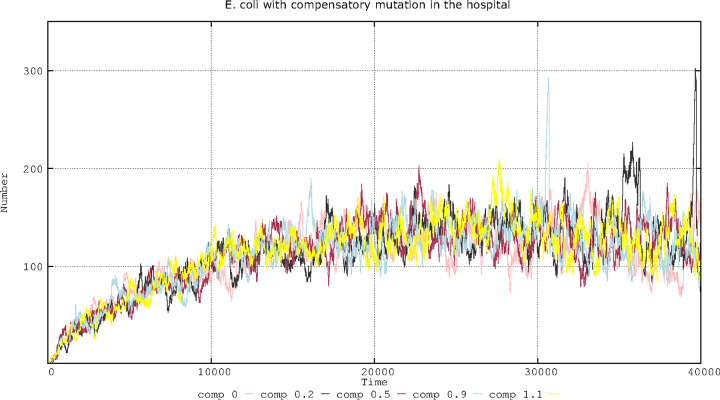
Effect of different compensation strengths of plasmid fitness cost on the number of cells with compensated plasmids. Lines are described as follows: 0, no compensation (pink); 0.2 cost compensation (black); 0.5 cost compensation (brown); 0.9 cost compensation (blue); 1.1 cost compensation (yellow). Numbers in ordinates correspond to hecto-cells (one unit = 100 cells in the microbiota) containing compensated plasmids. Note how little the strength of mutations compensating plasmid fitness cost influences the overall number of cells harboring compensated plasmids.

Mutation frequency should influence the emergence of plasmid cost compensatory mutations that can occur in several different genes. Compensatory mutations in the bacterial chromosome (41, 44, 45) and in the plasmid (46–49) might reduce the fitness cost of plasmid carriage, thus increasing the replication rate of the hosting microorganisms and the spread of plasmids. The emergence of compensatory mutations is certainly driven by the bacterial mutation frequency, but the effect of mutation might be asymmetrical if it occurs in the chromosome or plasmid given that mutated plasmids can horizontally disseminate more effectively than mutated chromosomes through vertical transmission and given the presence of several genes that can compensate the cost, which differs among bacterial hosts (47–54). In our basic model, high mutation frequency for plasmid cost compensatory mutations was expected to influence not only plasmid cost compensation but also the selection of chromosomal mutants (for instance, fluoroquinolone-resistant mutants). To separate the two effects in our model and to detect the effect of compensating for plasmid fitness cost, we increased the mutation frequencies but maintained the basic mutation frequency (10^−8^) for AbF*R observed for rifampin (55) that can be applied for AbF*R (56). In this model, plasmid fitness cost was set at 0.06, and the acquisition of a compensatory mutation decreases this value by one-half, 0.03. At a normal mutation frequency (10^−8^) or even 10^−5^ (data not shown), the effect of different strengths of mutational compensation on the frequency of plasmid-mediated antibiotic resistance was almost negligible in our model (Fig. 5).

**FIG 6:**
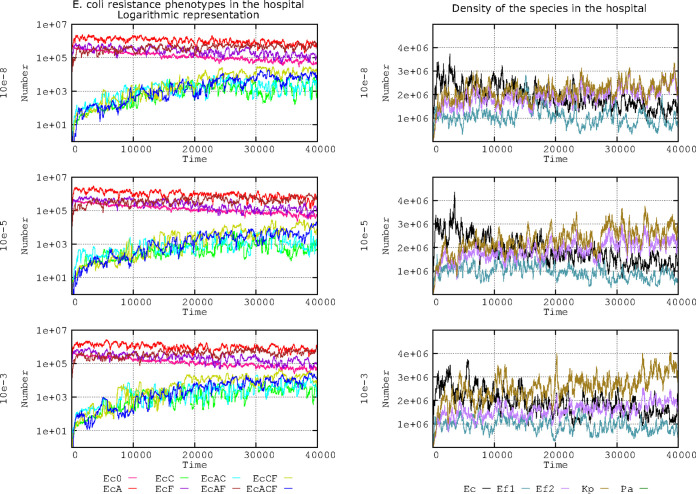
Influence of mutation frequencies providing compensatory plasmid fitness costs in E. coli resistance phenotypes and prevalence of bacterial species in the hospital. The left column shows the influence of different mutation frequencies (10^−8^, 10^−5^, and 10^−3^) compensating plasmid fitness costs in the evolution of E. coli resistance phenotypes. Ec0, susceptible to no resistance plasmids (pink line); EcA, PL1-AbAR (red); EcC, PL3-AbAR-AbCR (light fluorescent green); EcF, AbF*R (violet); EcAC, PL1-AbAR plus PL3-AbAR-AbCR (light blue); EcAF, PL1-AbAR plus AbF*R (brown); EcCF, PL3, AbAR-AbCR plus AbF*R (olive green); EcACF, PL1-AbAR plus PL3-AbAR-AbCR plus AbF*R (dark blue). The right column shows the corresponding effect on the species composition as follows: E. coli (black line), K. pneumoniae (dark olive green), ampicillin-resistant E. faecium (violet), ampicillin-susceptible E. faecium (blue green). Numbers in ordinates are expressed in hecto-cells (one unit = 100 cells in the microbiota). Note the very scarce consequences of the frequency of compensation of plasmid costs on the spread of plasmid-mediated resistant phenotypes.

Figure 6 shows the effect of the 10^−5^ mutation frequency (left column), which occurs by a small increase in E. coli lineages harboring compensated plasmids (olive green and dark blue lines), which is even more patent at the 10^−3^ mutation frequency. The reason for this small effect on E. coli can be explained by observing the overall landscape of the bacterial species included in the model (Fig. 6, right column); the proportion of K. pneumoniae containing a compensated plasmid increases with the mutation frequency, probably at the expense of E. coli and E. faecium. Note that high mutation frequencies allow bacteria with initially low population sizes to cross the mutational threshold to obtain a beneficial mutation (57). In summary, changes in mutation frequency for plasmid cost compensation does not influence or only slightly influences the long-term evolution of plasmid-harboring E. coli strains. Events with some epidemiological relevance are only expected to occur at poorly realistic mutation frequencies (10^−3^).

### Combined effects of compensation values for plasmid fitness cost and variation in plasmid fitness cost.

**FIG 7:**
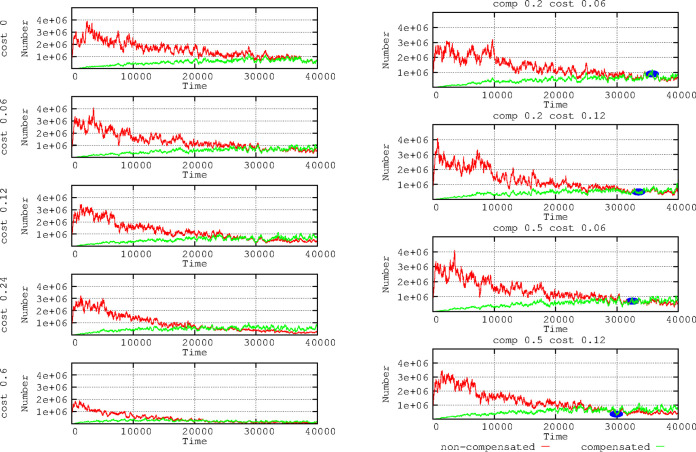
Effects of plasmid compensation depending on the plasmid fitness cost. In the left column, no compensated E. coli cells for plasmid fitness cost (green) versus compensated cells (red). In the right column, combinations of 2 fitness cost values (0.06 and 0.12) with 2 strength of compensation values (0.2 and 0.5). Small blue ovals highlight that when the plasmid cost is higher, the compensation is more effective (bacteria with compensated plasmids overcome the noncompensated). Numbers in ordinates are expressed in hecto-cells (one unit = 100 cells in the microbiota). Note that the higher the plasmid cost, the earlier cells with compensatory mutations overcome the noncompensated ones.

Figure 7 (left column; experiment with fixed mutation frequency for plasmid fitness cost compensation of 10^−5^) shows that the number of plasmid cost-compensated E. coli cells is lower when the cost of carrying the plasmid increases (given that bacteria replicate more slowly); however, cost-compensated bacteria (green line) outpace the noncompensated bacteria (red line) much earlier. Thus, the expected benefit of mutational compensation is proportional to the fitness cost imposed by the plasmid (48). By increasing the plasmid fitness cost, the replication of bacteria is impaired, thus possibly reducing the population size and the possibility of obtaining chromosomal compensatory mutations. Consequently, the reduction in plasmid fitness cost by compensatory mutations should increase population sizes. Conversely, the benefit of high mutation frequencies in reducing the plasmid fitness cost should be proportional to this cost. Figure 7 (right column) shows the outcome for our “hospital scenario” of the E. coli population with a cost-compensated and a noncompensated plasmidome. For the same level of compensation (0.2), the rise in compensated cells (green line) is higher for the 0.12 than for the 0.06 plasmid fitness cost. If we increase the fitness cost compensation level (to 0.5), the rise in mutated cells is even higher, as expected, because the mutational yield is proportional to the population size. In summary, the cost-compensated bacteria overcome earlier (in any case, it takes a long time) the noncompensated bacteria when the plasmid cost is high. However, at higher plasmid cost, the total number of bacteria decreases and, thus, the availability of compensatory mutants.

### Effect of changes in mutation frequency on combined fitness: compensation of plasmid fitness costs and acquisition of fluoroquinolone resistance.

**FIG 8:**
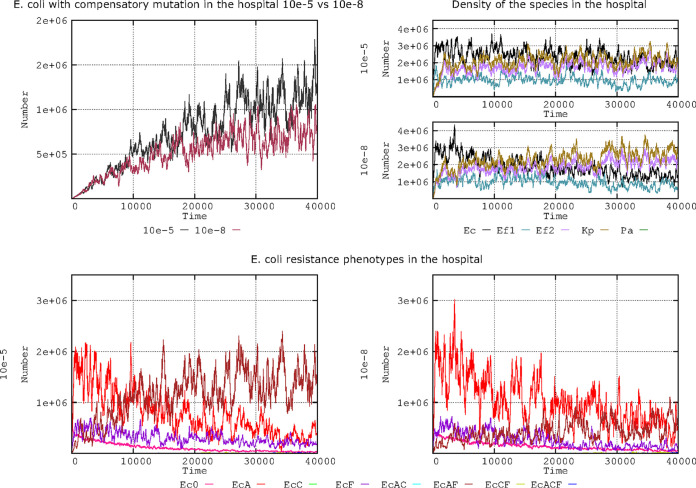
Compensation of plasmid fitness costs and frequency for fluoroquinolone resistance mutation. (Top left) E. coli cells compensated for fitness costs when mutation frequencies for fluoroquinolone resistance are 10^−8^ (black) or 10^−5^ (brown-violet). (Top right) The effect of these mutation frequencies on species distribution (E. coli [black line], K. pneumoniae [olive green], ampicillin-resistant E. faecium [violet], ampicillin-susceptible E. faecium [blue-green]). (Bottom) Evolution of E. coli antibiotic resistance phenotypes at fluoroquinolone mutation frequencies of 10^−5^ (left) and 10^−8^ (right). Note that at 10^−5^, the population combining PL1 (AbAR) (brown) maintains at long term a higher number of cells than the population with only PL1 (red) or AbF*R (violet). Numbers are expressed in hecto-cells (one unit = 100 cells in the microbiota). Note that increased mutation frequencies leading to more frequent fluoroquinolone resistance results in an increase of E. coli with cost-compensated plasmids, and both factors contribute to a long-term increase in E. coli populations.

In the natural world, we can consider that different mutation frequencies might influence various bacterial functions differently. For instance, fluoroquinolone resistance mutations in topoisomerases typically occur at a rate of 10^−8^, but the frequency of mutations influencing reductions in plasmid fitness costs might be much higher (e.g., 10^−5^) (51). In Fig. 8, there is a representation of the evolution in our complex landscape of the number of cells with plasmid cost compensation with a 10^−8^ or 10^−5^ fluoroquinolone resistance mutation frequency.

Statistically, cells with emerging mutations resulting in AbF*R rarely compensate the plasmid cost; however, once an AbF*R mutation selects an abundant population (58), there should be a higher probability that a compensatory mutation will emerge in this population that is able to reduce the plasmid fitness cost. Figure 8 (upper left column) shows the evolution of the number of cells in our complex landscape with plasmid cost compensation when the mutation frequency for fluoroquinolone resistance was 10^−8^ or 10^−5^. This result indicates that (as stated above), regardless of the mutational frequency for plasmid cost compensation, the increased survival of fluoroquinolone-resistant cells in the hospital environment increases the probability of mutation and, thus, the absolute number of plasmid cost compensatory mutants. In the 2 panels of the first column of Fig. 8 (right column), this effect is visible in the higher nosocomial prevalence of E. coli (black line) when AbF*R emerges at a frequency of 10^−5^. This strong increase in the AbAR-AbF*R population carrying PL1 (AbAR) in E. coli (brown line) when the mutation frequency is 10^−5^ is depicted in Fig. 8 (left column). The increase in cost compensatory mutations of PL1 in the increased AbAR-AbF*R population likely promotes its spread at these mutation frequencies, competing with the less frequent plasmid (PL3) and resulting in no clear advantage for AbCR (data not shown, available on request). In summary, increased mutation frequencies leading to more frequent fluoroquinolone resistance result in an increase of E. coli cells with cost-compensated plasmids, and both factors contribute to a long-term increase in E. coli populations.

## DISCUSSION

Plasmid biology should consider the multidimensional space where plasmids replicate and disseminate, not only inside and between bacterial cells but in complex ecosystems. Classic mathematical modeling approaches of plasmid maintenance and spread (27, 28) were based on mass-action ordinary differential equation (ODE) models, where the rate of an elementary interaction is proportional to the product of the concentrations of the interactive particles. However, these concentrations can be heterogeneous in spatially structured habitats, including biofilms in natural microbiotas. More recently, discrete-space continuous time stochastic models known as interacting particle systems (IPS) have been applied to study the spatial dynamics of plasmid transfer and persistence (59). An additional level of complexity is based on the inclusion in models of within-host and between-host modeling of plasmid spread (14).

The application of membrane computing models to study the horizontal conjugative transfer of antibiotic resistance genes in bacteria is one of the few available approaches for addressing bacterial evolutionary dynamics in such a broad nested ecological context (14). Based on our previously published model (12), in this study, the influence of various plasmid kinetic values in the evolution of antimicrobial resistance (8) was modeled within a complex system resembling the nested natural conditions that influence transmission at different levels (e.g., the flow of human hosts in the hospital and community, bacterial transmission/transfer rates among hosts, bacterial population sizes in the hosts, exposure and effects of various antibiotics in reducing bacterial numbers, selection of antibiotic-resistant species, and the influence of “space for colonization” of resistant strains in the microbiota) (12).

Details of the basic model’s design have been presented elsewhere (11–13). These integrative models are mostly fed with data on plasmid biology obtained through *in vitro* experiments and suggest that predictions based only on laboratory data may not necessarily reflect the evolution of resistance in natural clinical landscapes. This study presents only a model under particular conditions (see Materials and Methods), which were selected as representative examples; however, many other conditions can be introduced into the parameters in our accessible model (https://sourceforge.net/projects/ares-simulator/). Our main findings might help explain the relative weight of parameters that modify plasmid kinetics in the evolution of antibiotic resistance.

Plasmid transmission rates (the conjugation rates in our model) have been considered one of the main drivers of the spread of antibiotic resistance genes in natural bacterial populations (38). In fact, there is a line of research on ecology-evolution (eco-evo) drugs that seeks to develop plasmid conjugation inhibitors to reduce the burden of antibiotic resistance (60, 61). In our complex multilevel system and under the fixed conditions of the simulation, only high (10^−3^) conjugation rates clearly influence the dissemination of plasmids and the resistances they contain. The results obtained in mass-action models conclude that the spread of resistance depends only in a subtle way on the rates of gene transfer (62). However, interacting particle system models detect a significant influence of conjugation rates on plasmid maintenance and spread when the spatial structure is considered (59). In fact, we were also able to differentiate between conjugation rates of 10^−6^ (in which the plasmid PL3 slowly propagates) from 10^−9^ (in which effects or transfers are no longer visible). Interestingly, at higher conjugation rates (at which E. coli transfers PL1 [AbAR] effectively), PL1 tends to displace PL3 (AbCR, AbAR) resistance in K. pneumoniae. Thus, the PL3 is only preserved in K. pneumoniae under low E. coli conjugation rate conditions.

Plasmid compatibility is dependent on the total number of plasmids sharing the same *par* system that can coexist in the same cell. The number of low-copy-number plasmids in a cell is controlled by active partitioning systems but influenced by cell metabolism and host-cell cycle, as plasmid and host cell cycles are not synchronized, as it was shown in theoretical kinetic studies (63, 64). In our simulation, we consider two related plasmids (PL1 and PL3) with different replicons that are, therefore, able to coexist in the same cell if a tolerated copy number is not surpassed. Under conditions allowing only a single plasmid to be maintained, PL3 (containing AbCR-AbAR) from K. pneumoniae can displace PL1 (AbAR); thus, AbCR significantly increases in E. coli. Conversely, the introduction of PL1 from E. coli into K. pneumoniae severely reduces the number of K. pneumoniae cells harboring PL3. In the conditions set in the present simulation (conjugation rate of 10^−6^), if the cell tolerates two copies and PL1 + PL3, the number of E. coli cells harboring PL3 (AbCR) comparatively decreases, and there are no significant differences if the cell is able to maintain three plasmid copies, a result that might be modified by the conjugation rate. In a previously published study by our group (12), K. pneumoniae retained PL3 (AbCR) if the conjugation rate is higher, 10^−4^. Note that in the real world, K. pneumoniae frequently serves to introduce into the ecosystem plasmids with AbCR that are then transferred to E. coli and subsequently among E. coli populations (65, 66). Because E. coli has a comparatively higher population size and most of the conjugations occur at the intraspecies level, in many cases, AbCR hospital “epidemics” tend to occur over a long term in E. coli, and AbCR K. pneumoniae is usually maintained at a lower frequency (67–70).

Plasmids segregate (disappear, are lost) from the cells in which they are hosted, but the segregation rates are not well established and depend on the ecological-physiological conditions of the bacteria and the segregation mechanisms. In our model system, plasmid loss rates of 10^−3^ led to plasmid extinction. In general, high segregation rates favor plasmids contained in large bacterial populations. At 10^−4^, for example, only PL1 contained in the dominant E. coli population persists in time, displacing populations with PL3 despite the stronger selection pressure (due to AbCR). Higher exposure to antibiotics (cephalosporins) selecting for PL3 should logically favor the maintenance of PL3. In any case, PL3 persists over the long term much more effectively at low segregation levels (such as 10^−5^ and 10^−6^). In the present version of our model, the role of toxin-antitoxin postsegregational killing mechanisms in plasmid stabilization, eventually in synergy with spatial differentiation (biofilms), was not addressed (71, 72), but we are signaling here the potential importance of this process in plasmid persistence and spread.

Plasmid fitness costs, expressed as a reduction in bacterial growth rate, have a relevant influence on bacterial resistance phenotypes, particularly on the propagation of plasmids hosted by minority populations. This effect is particularly visible when the plasmid cost is ≥0.12. For instance, the spread of PL3 (with AbCR) primarily hosted by K. pneumoniae is strongly reduced beyond a cost of 0.12. Even if the same cost was imposed by PL1, as is frequently present in the dominant E. coli, the reduction in fitness is somewhat compensated by intraspecific transfer; however, when the fitness costs is 0.60, the population with PL1 steadily decreases. Although AbF*R populations are not influenced by plasmid fitness costs, many of the AbF*R cells are lost because of the reduced reproductive rate imposed by the cost of plasmids they may contain.

Mutations might compensate for the plasmid fitness costs. The mutation frequency should, therefore, affect the number of cost-compensated plasmids, particularly the plasmids imposing a higher fitness cost. In our model, the effect on plasmid-mediated resistance was almost undetectable at the “consensus” mutation frequency (10^−8^), which might suggest that this compensation parameter has low epidemiological consequences. Even at a general mutation frequency of 10^−5^, which occurs in mutator strains (55), the effects on the spread of antibiotic resistance under our experimental conditions were barely detectable. However, we cannot reject the possibility of scenarios in which the mutational cost compensation of E. coli lineages harboring plasmids tends to increase (73). In fact, this might explain why E. coli isolates harboring plasmids with extended-spectrum β-lactamases have increased mutation frequencies (74). As proof of this concept, the benefit for cost-compensated strains (and for K. pneumoniae) becomes clear in a hypothetical scenario containing strains with a 10^−3^ mutation frequency.

In principle, the beneficial effect of plasmid cost compensation on the evolution of plasmid spread should be proportional to the reduction in fitness imposed by plasmid carriage. On one hand, our simulation shows that the cost-compensated bacteria surpass the number of noncompensated bacteria earlier when the cost is high. On the other hand, the number of plasmid cost-compensated E. coli cells decreases when the cost of carrying the plasmid increases, given that the availability of mutants is dependent on the population size.

Given that increases in general mutation frequency should necessarily influence the emergence of chromosomal fluoroquinolone resistance mutations (AbF*R), we combined the effects of the mutation frequency on AbF*R acquisition and plasmid cost compensatory mutation. Our results indicate that, regardless of the mutational frequency for plasmid cost compensation, the increased survival and population increase of fluoroquinolone-resistant cells with high mutation frequencies increase the absolute number of plasmid cost compensatory mutants.

Note that the results of this study correspond to a limited number of possible parametric landscapes; however, our intention was to use the parameters that frequently influence plasmid and bacterial dissemination in hospital settings. Further developments of our model will include more detailed spatial heterogeneities to detect the effect of possible pockets of plasmid-containing local populations (59), which were not considered in this version because of the current limitation in the computational load. In any case, a major advantage of the membrane computing modeling technology is its scalability, allowing us to include many different parametric values simultaneously in the model at the various hierarchical levels considered in particular ecosystems in workload and scope. How changes in a “piece” (the plasmid) contribute to create a particular “pattern” in a nested system of biological units, expanding from the genes and cells to the communities of human hosts and environments, is certainly one of the challenges of modern research on antibiotic resistance (6, 75, 76).

## MATERIALS AND METHODS

### Computing model.

All computational simulations were performed using an updated version of the Antibiotic Resistance Evolution Simulator (ARES), which is a P system software implementation for modeling antibiotic resistance evolution (11, 13). As stated in Results, the P system approach is stochastic in nature because of some of the elements it incorporates; on the one hand, the rules governing the model’s behavior incorporate execution probabilities of a biological nature that serve as a measure of the propensity of each rule. On the other hand, the probabilistic execution of rules considers the computation until each moment of its application. Therefore, the computation that executes the model could be considered a stochastic source in a stationary state given that the propensity constants do not evolve temporarily, but it is the biological nature of the interactions between computational “objects” within and between hierarchical levels itself that fixes them. The current version of ARES (2.0) can be freely downloaded (https://sourceforge.net/projects/ares-simulator/). The original ARES website (http://gydb.uv.es/ares) offers information on the rules and parameters currently used by ARES and facilitates customer generation of specific scenarios to model the evolution of antibiotic resistance.

### Basic model application: quantitative structure.

A detailed account of the main features of our model’s quantitative structure is available in our previous publication (12). A single scenario with a fixed number of values was used in this work, representing reasonable (but not necessarily precise) numbers in the real world, a summary of which is presented below. Note that all of these values can be changed in the model, and the results are expected to differ according to the different scenarios.

### Hospitalized hosts, admissions, and discharge rates in the population.

The number of hosts in the hospital reflects an optimal proportion of 10 hospital beds per 1,000 individuals in the community (https://data.oecd.org/healtheqt/hospital-beds.htm). The hospital has 100 occupied beds and corresponds to a population of 10,000 individuals in the community. The admission and discharge rates from hospital are equivalent to 3 to 10 individuals/10,000 population/day (https://www.cdc.gov/nchs/nhds/index.htm). In the basic model, 6 individuals from the community are admitted to the hospital and 6 are discharged from the hospital to the community per day (approximately at 4-h intervals). Approximately 75% of the patients stay in the hospital between 6 and 9 days.

### Transfer of bacterial organisms between hospitalized hosts.

We used a “contagion index” of 5% (for every 100 hospitalized patients, 5 “donors” transmit bacteria to another 5 “recipients” per hour). Bacterial transmission includes the spread of normal microbiota. The hospital is surrounded by a community of healthy individuals, occasionally admitted to the hospital, with a “contagion index” of 0.01%.

### Exposure to antibiotic agents.

We considered 3 types of commonly used antibiotics to be employed during a 7-day treatment as follows: aminopenicillins (AbA), third-generation cephalosporins such as cefotaxime (AbC), and fluoroquinolones (AbF). In the basic model, 20% of the individuals in the hospital compartment are under antibiotic exposure each day. Antibiotics AbA-AbC-AbF are employed in the hospital at a proportion (percentage) of 30:40:30, respectively. A single patient is treated with only one antibiotic, administered every 8 h. After each dose is administered, all 3 (bactericidal) antibiotics induce after a decrease of 30% in the susceptible population after the first hour of dose exposure and a 15% reduction in the second hour. In the community surrounding the hospital, 1.3% of individuals are undergoing antibiotic therapy; AbA-AbC-AbF is employed at a proportion of 75:5:20, respectively.

The bacterial colonization space is the volume of the colonic space occupied by bacterial populations. These populations include clinical species (E. coli, K. pneumoniae, Pseudomonas aeruginosa, Enterococcus faecium) and other microbiota populations. In natural conditions, the sum of these populations is estimated at 10^8^ cells per milliliter of colonic content. Clinical species constitute only 1% of the cells in each milliliter and have a basal colonization space of 1% of each milliliter of colonic content (or 0.01 ml). Other microbiota populations are considered a single ensemble. The colonic space occupied by these populations can change due to antibiotic exposure. AbA, AbC, and AbF reduce the intestinal microbiota 25%, 20%, and 10%, respectively. This space can be occupied by resistant populations of these human opportunistic pathogens; however, in the absence of antibiotic exposure, the colonic populations tend to return to the basal population size, which would occur in 2 months (77, 78).

### Population operative packages and counts.

To facilitate the execution of the model, we considered that 10^8^ cells in nature is equivalent to 10^6^ cells in the model. In other words, one “hecto-cell” (h-cell) in the model is an “operative package” of 100 cells in the real world. Given the high effective population sizes in bacteria, these 100 cells are considered a uniform population of a single cell type. For computational efficiency, we considered that each patient (in the hospital) or individual (in the community) is represented in the model by 1 ml of its colonized colonic space (approximately 3,000 ml) and is referred to as a “host-ml”. Our results are, therefore, represented as “number of h-cells in all host-mls” in most of the figures.

### Quantitative distribution of species and clones.

In the basal scenario, the species distribution in these 1,000,000 cells (contained in 1 ml) was as follows: for E. coli, 860,000 cells, including 500,000 susceptible cells, 250,000 containing PL1-AbAR, 100,000 with the AbF*R mutation, and 10,000 with the AbF*R mutation and carrying PL1 (AbAR); for E. faecium, 99,500 cells susceptible to both AbA and AbF and 20,000 cells with chromosomal AbCR, AbF*R, and CO1-AbAR (CO for *Enterococcus* conjugative element); for K. pneumoniae, 20,000 cells with chromosomal AbAR and AbF*R also harboring PL3 (AbCR-AbAR); and P. aeruginosa, 500 cells containing PL3 (AbCR-AbAR) and chromosomal AbAR. At time zero, this distribution was identical in hospitalized and community patients.

### Bacterial multiplication rates.

We considered the basal multiplication rate (corresponding to E. coli 0) to be equal to 1, in which each bacterial cell gives rise to 2 daughter cells every hour. Comparatively, the rates for E. faecium, K. pneumoniae, and P. aeruginosa were 0.85, 0.9, and 0.15, respectively. In these basic conditions, the acquisition of a plasmid or other incurs a cost of 0.06, while the acquisition of the AbF*R mutation incurs a cost of 0.01 (1% reduction in growth rate). The number of cell replications will be limited by the available space (see above).

### Plasmids and antibiotic resistance types.

For the sake of simplicity and inspired by the high frequency of IncF plasmids involved in the spread of beta-lactamases in the hospital setting, this study considers two related plasmids carrying multiple replicons and, thus, able to coexist in the same cell (34). These plasmids contain genes encoding (i) resistance to AbA (A for aminopenicillins) present in plasmid PL1 and primarily hosted in E. coli, or (ii) resistance to antibiotic C plus antibiotic A (AbC; C for third-generation cephalosporins) primarily determined by the plasmid PL3 and primarily hosted in K. pneumoniae. Note that some widely spread plasmids that encode extended-spectrum beta-lactamases (ESBLs) frequently carry genes for AbA resistance. In addition, mutational events lead to the emergence of chromosomal fluoroquinolone resistance (AbF; F for fluroquinolones). Organisms mutate to AbF at the same rate as follows: 1 mutant for every 10^8^ bacterial cells per cell division. Note that K. pneumoniae has intrinsic (plasmid-independent) resistance to AbA. Although it was not analyzed in this study (but is included in the model), a clone of E. faecium resistant to AbA might transfer this resistance to a susceptible clone at a rate of 10^−4^ (79).

### Basal plasmid kinetic values.

We established a basal set of plasmid kinetic values based on reports in the literature and referenced in the previous paragraphs. In the simulation, modifications to each value were applied and maintaining the other values fixed; ultimately, more than one parameter was modified to ascertain the combined effects. The applied basal plasmid kinetic values were as follows: (i) a plasmid transfer rate of 10^−6^ per hour, i.e., 1 in 1 million donor-recipient contacts that result in random and reciprocal (not in the same conjugative event) cell-to-cell E. coli-K. pneumoniae transfer of the plasmid (10^−9^ from each of them to P. aeruginosa) every hour; (ii) a tolerated plasmid copy number of 2 plasmids/cell, indicating that, in the presence of a third plasmid, one of the three is stochastically removed; (iii) a rate of plasmid cost of 0.06 (i.e., the bacterial growth rate is decreased by 6% when harboring plasmids); (iv) a rate of frequency of mutational plasmid cost compensation of 10^−5^ (i.e., in 1 per 100,000 cells, a mutational event in the plasmid or bacterial genome decreases the plasmid cost by 50%); (v) a rate of mutational events leading to significant fluoroquinolone resistance of 10^−8^; and (vi) a rate of plasmid segregation of 10^−5^, indicating that stochastically 1 cell among 100,000 eliminates the plasmid it contains.

## Supplementary Material

Supplemental file 1
