# Supplementary material for: Simulating the Influence of Conjugative-Plasmid Kinetic Values on the Multilevel Dynamics of Antimicrobial Resistance in a Membrane Computing Model
Source: Antimicrob Agents Chemother. 2020 Jul 22;64(8):e00593-20. doi: 10.1128/AAC.00593-20 (PMC7526830; doi:10.1128/AAC.00593-20)
Supplement: Supplemental file 1 [file AAC.00593-20-s0001.pdf]

Supplemental Material

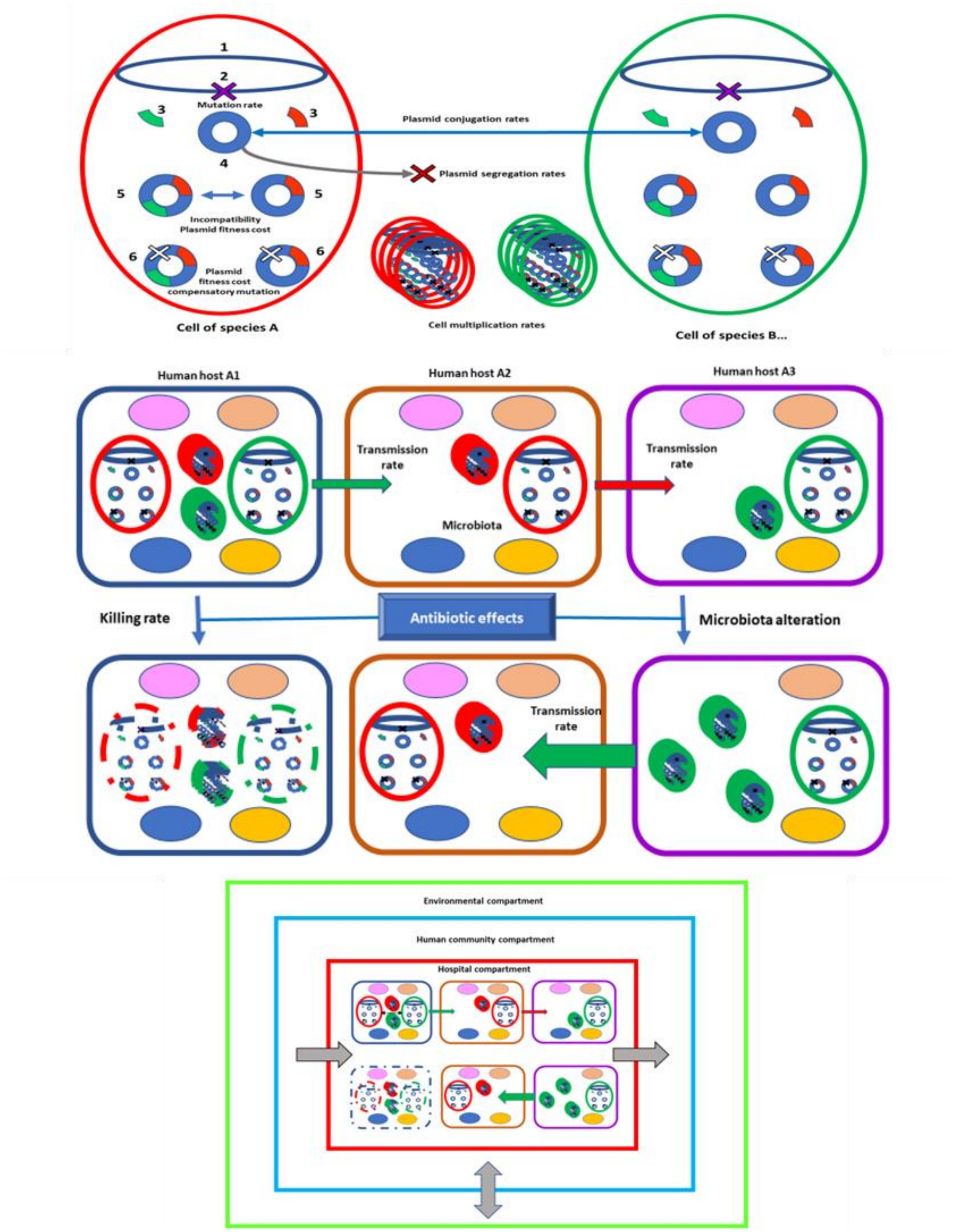

Figure S1. Simplified graphic schema of the membrane computation model. On the top panel, two bacterial cells of the same or different species (red and green circles) containing a

chromosome (black oval, 1), where mutations might occur at variable rates (black X, 2); the absolute number of these cells might be modified. Inside each cell, different antibiotic resistance genes (red, green, 3), that might be harbored in plasmids of the same Inc groups (blue circle crown, 4). Plasmids might compete inside the cell, as there is a fixed copy-number per cell, eventually leading to the segregation of one of these plasmids; also plasmids might be submitted to random loss (X, 4), or impose a fitness cost to the host cell (5); however, cost-compensatory mutations can reduce this cost, restoring in part or totally the host-cell replication rate (6). Cells can replicate at different rates (cylinders of red or green ovals). Plasmids can be transferred between cells at different conjugation rates (horizontal black line). On the middle panel, each one of the squares with curved angles correspond to a different human hosts (different colors) where these bacterial cells are established; the colored ovals inside each host correspond (in a simplified way) to the different species in the microbiota. Bacteria harboring plasmids with resistance genes can be transferred from human to human hosts at variable rates (for instance, influenced by hospital hygiene or cross infection). Submitted to antibiotic exposure, different antibiotics can kill (eliminate) bacterial cells at certain rates, but bacteria might survive is they have resistance genes; note that other bacteria of the microbiota can also be eliminated by antibiotics, eventually increasing the population size of the surviving resistant bacteria (as the green cylinders down right), which can be transferred to new hosts. In the lower panel it is depicted that all these processes can occur inside a hospital (red square), or in the human community where the hospital is located (blue square); these compartments are linked by variable admission and discharge rates (horizontal grey arrows) that can also be introduced in the model; finally, the bacterial composition inside the human community is influenced by the interaction with the environment (green square). This figure reveals the multi-nested structure of units involved in antibiotic resistance.
